# Altered Ca^2+^ Homeostasis and Endoplasmic Reticulum Stress in Myotonic Dystrophy Type 1 Muscle Cells

**DOI:** 10.3390/genes4020275

**Published:** 2013-06-04

**Authors:** Annalisa Botta, Adriana Malena, Emanuele Loro, Giulia Del Moro, Matteo Suman, Boris Pantic, Gyorgy Szabadkai, Lodovica Vergani

**Affiliations:** 1 Department of Genetics, University “Tor Vergata”, Roma 00133, Italy; E-Mail: botta@med.uniroma2.it; 2 Department of Neurosciences SNPSRR, University of Padova, Padova 35100, Italy; E-Mails: adriana.malena@unipd.it (A.M.); delmoro.giulia@gmail.com (G.D.M.); boris.pantic@yahoo.it (B.P.); 3 Department of Physiology, Pennsylvania Muscle Institute, University of Pennsylvania, Philadelphia, PA 19104, USA; E-Mail: eloro@mail.med.upenn.edu; 4 Department of Biomedical Sciences, University of Padua and CNR Neuroscience Institute, Padua 35100, Italy; E-Mails: matteo.suman@gmail.com (M.S.); g.szabadkai@ucl.ac.uk (G.S.); 5 Department of Cell and Developmental Biology, Consortium for Mitochondrial Research, University College London, London WC1E 6BT, UK

**Keywords:** myotonic dystrophy, muscle cells, Ca^2+^ homeostasis, SERCA, Ryr1, Cav1.1, ER stress

## Abstract

The pathogenesis of Myotonic Dystrophy type 1 (DM1) is linked to unstable CTG repeats in the *DMPK* gene which induce the mis-splicing to fetal/neonatal isoforms of many transcripts, including those involved in cellular Ca^2+^ homeostasis. Here we monitored the splicing of three genes encoding for Ca^2+^ transporters and channels (RyR1, SERCA1 and CACN1S) during maturation of primary DM1 muscle cells in parallel with the functionality of the Excitation-Contraction (EC) coupling machinery. At 15 days of differentiation, fetal isoforms of SERCA1 and CACN1S mRNA were significantly higher in DM1 myotubes compared to controls. Parallel functional studies showed that the cytosolic Ca^2+^ response to depolarization in DM1 myotubes did not increase during the progression of differentiation, in contrast to control myotubes. While we observed no differences in the size of intracellular Ca^2+^ stores, DM1 myotubes showed significantly reduced RyR1 protein levels, uncoupling between the segregated ER/SR Ca^2+^ store and the voltage-induced Ca^2+^ release machinery, parallel with induction of endoplasmic reticulum (ER) stress markers. In conclusion, our data suggest that perturbed Ca^2+^ homeostasis, via activation of ER stress, contributes to muscle degeneration in DM1 muscle cells likely representing a premature senescence phenotype.

## 1. Introduction

Myotonic dystrophy (Steinert’s disease, 1909) is the most prevalent form of adult muscular dystrophy with a frequency of 1 in 8,000 individuals worldwide [1]. Type 1 (DM1, MIM160900), also called Steinert’s disease [2], has a severe congenital form and a milder adult-onset form. Affected individuals express highly heterogeneous, multisystemic symptoms including myotonia, progressive muscle weakness and wasting, cataracts, hypogonadism, frontal balding, cardiac conduction defects and ECG changes [3]. DM1 is a progressive disease and its symptoms become more severe with age and across generations. The genetic defect in DM1, identified in 1992, results from the expansion of a CTG repeat in the 3' untranslated region (UTR) of dystrophia myotonica protein kinase (DMPK. MIM #605377), a gene which maps to the chromosome 19q13.3 and encodes a serine/threonine protein kinase [4]. Unaffected individuals have <38 CTG repeats, whereas expansions associated with DM1 range from 80 to >2,500 repeats. Repeat length correlates directly with disease severity and inversely with the age of onset [5]. Amplification is frequently observed after parent-to-child transmission, but extreme amplifications are not transmitted through the male line. This mechanism explains genetic anticipation [6,7] and the occurrence of the severe congenital form almost exclusively in the offspring of affected women [3].

Different histopathological changes occur in the DM1 muscle, including predominance and atrophy of type I fibers, augmented incidence of ring fibers and variability in fiber size, increased number of central nuclei and sarcoplasmic masses [8]. Other studies reveal abnormalities of endoplasmic reticulum (ER), dilatation of the terminal cisternae [1] and sarcoplasmic masses associated with stress markers of the endoplasmic reticulum (ER) [9], indicators of a perturbed physiological function of ER.

Nuclear aggregates of mutant DMPK mRNA and proteins (foci), identified by RNA fluorescence *in situ* hybridization (RNA-FISH), sequester two splicing regulators implied in the development of the great majority of DM1 symptoms. The misregulated alternative splicing, or spliceopathy, is linked both to increased levels of CUG binding protein 1 (CUG-BP1) [10] and sequestration of Muscleblind-1 like protein (MBNL1) [11].

The altered levels of CUG-BP1 and MBNL1 result in reversion to embryonic splicing pattern of several mRNAs, such as the muscle chloride channel *CLCN1* and insulin receptor *INSR*, resulting in myotonia and insulin resistance [4,12].

Dysregulated Ca^2+^ homeostasis is generally considered a key initiator of muscle degeneration in DM1. Intracellular Ca^2+^ is an important regulator of both cell proliferation and apoptosis. Defects in Ca^2+^ homeostasis have been associated with many pathological conditions such as increased death of cardiomyocytes in Duchenne muscular dystrophy [13]. Under stress conditions, Ca^2+^ transfer from the ER to the mitochondria can result in Ca^2+^ overload in the mitochondrial matrix, triggering permeability transition (mPT). mPT results in collapse of the mitochondrial membrane potential, increased reactive oxygen species (ROS) production and release of pro-apoptotic factors (e.g., cytochrome c and apoptosis-inducing factor) [14,15].

In skeletal muscle cells excitation-contraction coupling (ECC) and regulation of intracellular Ca^2+^ homeostasis is mediated by the coordinated action of a set of Ca^2+^ channels and transporters: Cav1.1, ryanodine receptor 1 (RyR1) and sarcoplasmic/endoplasmic reticulum Ca^2+^-ATPase (SERCA). Cav1.1 (DHRP_α1S_), is an l-type calcium channel and voltage sensor, located in triad junction in close proximity to RyR1. In response to plasma membrane depolarization, Cav1.1 physically interacts with RyR1, which in turn releases Ca^2+^ from the SR. Ca^2+^ is subsequently pumped back into the lumen of SR by SERCA to allow relaxation. As a consequence of SERCA activity, the resting cytosolic free [Ca^2+^] is maintained three to four orders of magnitude lower than the intra-SR/ER [Ca^2+^] [16].

Numerous works have been focused on the effects of the DMPK gene (CTG)_n_ expansion on the splicing [17,18,19], expression [20,21] and functionality [19,20,21,22] of Cav1.1, RyR1 and SERCA during the last 20 years. There are three genes encoding RyR in mammals: RyR1 and RyR2 are expressed in skeletal muscle and heart, respectively, while RyR3, although it is not predominant, is detected in immature skeletal muscle and markedly decreases at later stages of development. The expression of RyR1 splice variants is regulated both developmentally and in a tissue-specific manner [23]. The variant ASI(−), which lacks ASI (exon 70, residues 3481–3485), constitutes 100% of RyR1 expressed in mouse embryonic skeletal muscle which decreases to 30% in the adult muscle. Another splicing variant, ASII(−), which lacks ASII (exon 83, residues 3865–3870), is transiently expressed in skeletal muscle after birth and constitutes 10% of RyR1 in adults. In mice, the splicing change in ASII takes place earlier than that in ASI and the ASII(+) isoform is dominantly expressed even in human myotubes [17]. SERCAs are also encoded by three homologous genes: SERCA1, SERCA2 and SERCA3. Transcripts from these genes undergo alternative splicing in a developmentally regulated and tissue-specific manner, giving rise to isoforms that differ in seven amino acids in their *C*-terminal region. SERCA1a (adult form) and 1b (neonatal form) are mainly expressed in fast-twitch (type 2) skeletal muscle and SERCA2a is expressed in slow-twitch (type 1) skeletal and cardiac muscles, whereas SERCA2b expression is ubiquitous. SERCA3 is expressed in several non-muscle tissues at variable levels, co-localizing with SERCA2b. At mRNA level, the fetal isoforms of both transporters are increased in DM1 muscle, causing a documented alteration of Ca^2+^ homeostasis [17,18]. More recently, it has been proposed that muscle weakness could also be associated with a misregulated alternative splicing of CACNA1S, the gene encoding Ca_v_1.1, the l-type Ca^2+^ channel and voltage sensor that plays a central role in excitation-contraction coupling [19]. 

However, the exact molecular mechanism responsible for muscle weakness and wasting in DM1 remains largely unknown. Interestingly, a close relationship between dysregulated ER/SR Ca^2+^ homeostasis and the ER stress response has been recently described. ER stress is induced by the accumulation of unfolded proteins in the ER lumen, due to various stress *stimuli* impinging on the normal folding machinery. Protein folding in the ER involves Ca^2+^ dependent steps, mediated by Ca^2+^ binding chaperones, such as grp78 (BiP), thus deregulating the ER/SR Ca^2+^ homeostasis could directly trigger protein misfolding. On the other hand, the transcriptional response to the ER stress involves altered expression of components of the ER Ca^2+^ handling machinery. Ultimately, ER stress can induce mitochondrial Ca^2+^ overload, where mPT activation leads to bioenergetic crisis and different modalities of cell death. 

Our group recently showed that primary DM1 myotubes present an interruption of late myogenic program coincident with an increase of apoptotic/autophagic cell death and oxidative stress [24] after 12–15 days of differentiation. The contribution of programmed cell death to the muscle wasting typical of DM1, rather than altered myogenesis, poses new questions about the development of the muscle pathology. To better understand the link between the premature senescence and impaired regeneration and maintenance of muscle mass, we studied cellular and ER/SR Ca^2+^ homeostasis, splicing and protein levels of Ca^2+^ transporters in parallel with ER stress markers in affected mature muscle cells.

## 2. Materials and Methods

*Skeletal muscle biopsies*. After written consent, DM1 (n = 5) muscle samples were obtained from vastus lateralis by diagnostic open biopsies. Control samples (also from vastus lateralis, n = 5) were obtained from subjects deemed free of neuromuscular disorders. Molecular diagnosis of DM1, using a combination of long-PCR and Southern blot analysis in peripheral blood, showed that DM1 patients considered in this study had a CTG expansion between 800–1,500 repetitions [25].

*Cell culture*. Cells were cultured in Ham’s F14 medium (Euroclone) plus 30% FBS (Gibco) and 10 μg/mL insulin (Sigma). When myoblasts reached a 70% of confluence, differentiated myotubes were obtained by reducing the FBS in the medium at 2%. Samples were collected at 10 and 15 days of differentiation. 

*RNA extraction, RT-PCR analysis*. Total RNA was extracted from frozen muscle biopsies and from 10–15 days differentiated myotubes using Trizol^®^ reagent (Sigma). 1 μg of total RNA was reverse-transcribed to cDNA with SuperScript^®^ III First-Strand Synthesis System (Invitrogen). The PCR splicing assays for RyR1 [ASI F: 5' GACAACAAAAGCAAAATGGC 3'; R: 5' CTTGGTGCGTTCCTGGTC 3'; ASIIF: 5' TTGAGAGACAGAACAAGGC 3'; R: 5' GGTCTTGTGTGAATTCATCA 3']; SERCA1 [F: 5' ATCTTCAAGCTCCGGGCCC 3'; R: 5' CAGCTCTGCCTGAAGATGTG 3']; CACNAIS gene [F: 5' ATGTGTTTGACTTCCTGATT 3'; R: 5' GTAGATGAAGAAGAGCATGAC 3'] were performed as previously described [26]. Total PCR products, obtained within the linear range of amplification, were electrophoresed on 3.5% agarose gel. Quantitative analysis of the amplified products was performed using SyberGreenII (Life Science) staining of the gel. The integrated optical density of each band and the fraction of fetal *vs.* total transcript were quantified by densitometry using commercial software. The expression levels of CHOP [F: 5' AGCTGAGTCATTGCCTTTCT 3'; R: 5' CTGGTTCTCCCTTGGTCTTC 3'] and GRP78 [F: 5' GGCCGCACGTGGAATG 3'; R: 5' ACCTCCAATATCAACTTGAATGTATGG 3'], compared to the expression of β-actin [F: 5' GACAGGATGCAGAAGGAGATTACT 3'; R: 5' TGATCCACATCTGCTGGAAGGT 3'], were quantified by Sybergreen RT-PCR using the ABI PRISM7000 sequence detection system.

*Ca^2+^ measurements*. T10-15 myotubes were seeded on glass coverslips, and loaded with the fluorescent Ca^2+^ sensitive probe Fura2-AM (5 μM at 37 °C, 30 min). [Ca^2+^] was assessed by calculating the ratio of fluorescent intensities measured at 340 nm and 380 nm excitation and 520 nm emission on Olympus IX81 inverted fluorescence microscope as previously described [27]. Membrane depolarization was induced by the addition of 120 mM KCl at 37 °C in isotonic extracellular solution (10 mM NaCl, 120 KCl, 1 mM MgSO_4_, 5.5 mM glucose, 20 mM HEPES, pH 7.4) as previously described [28]. Single traces obtained for each myotube were analyzed with commercial software. Traces were quantified to determine the peak Ca^2+^ response, the area under the curves (AUC) for statistical analysis.

*Immunofluorescence analysis*. Immunofluorescence analysis of SERCA2 and of RyR1 was performed on fixed myotubes, permeabilized with 0.2% Triton X-100 and incubated for 30 min with 0.5% BSA and 10% horse serum in PBS. Primary specific antibodies were diluted in PBS plus 2% BSA and incubated overnight at 4 °C (SERCA2, Santa Cruz and RyR1, Thermo Scientific). Secondary Alexa488 or Cy3-conjugated antibodies (Invitrogen-Molecular Probes) were incubated for one hour. Samples were mounted in Vectashield mounting medium with DAPI (4'-6-diamidino-2-phenylindole) (Vector Laboratories) and observed on Olympus BX60 fluorescence microscope

*Western Blot analysis.* Myotubes were collected after 10 and 15 days of differentiation. For muscle samples, 10 µm cryosections were used. Total protein extracts were electrophoresed on 7.5–17.5% T30C4 SDS-PAGE gels or 4–12% NuPAGE precast gels (Invitrogen). Proteins were blotted onto nitrocellulose membrane or 0.45 µm PVDF (Invitrogen) and probed with specific antibodies against SERCA 2 (Santa Cruz), RyR1 (Thermo Scientific), DHRP_α1S_ (Thermo Scientific), calsequestrin (Thermo Scientific), calnexin (BD Biosciences), CHOP (Santa Cruz), GRP78 (BD Biosciences), MG29 (Santa Cruz), β-actin (Chemicon). After incubation with specific secondary HRP-conjugated antibodies, recognized bands were visualized by chemiluminescence (GE HealthCare). Integrated optical density of each band was calculated with commercial software and normalized compared to β-actin.

*Statistical analysis.* Quantitative data are presented as mean ± SD. In the case of normal distribution of values, confirmed by Shapiro’s test, statistical comparisons were performed using the Student’s *t* test. Values of *p* < 0.05 were considered significant.

## 3. Results

### 3.1. Cellular and ER/SR Ca^2+^ Homeostasis Is Altered in Primary DM1 Myotubes

Primary DM1 myoblasts are capable of differentiating into myotubes. However, DM1 myotubes display increased ROS production, autophagy and induction of apoptosis [24] after 10–15 days of differentiation. To determine whether altered Ca^2+^ homeostasis plays a role in triggering cellular dysfunction, primary cultures of muscle cells were established from biopsies of five DM1 patients and five age-matched healthy individuals. In order to induce differentiation of myoblasts into myotubes, we cultured cells in differentiation medium for either 10 days (T10), when programmed cell-death started, and for 15 days, when the majority of myotubes are committed to cell death [24]. 

**Figure 1:**
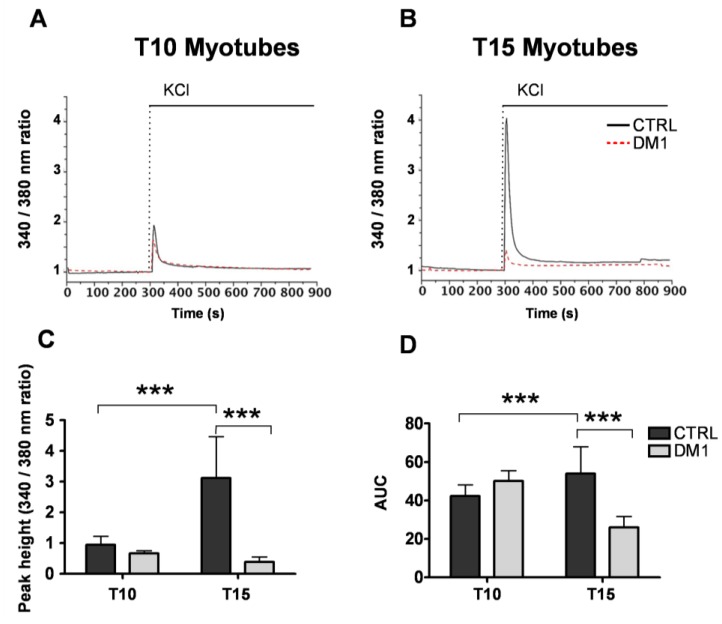
Depolarization induced cytosolic Ca^2+^ responses in control and Myotonic Dystrophy type 1 (DM1) myotubes at sequential stages of differentiation. Depolarization was induced by the addition of 120 mM KCl to the myotubes at 10 days (T10, **A**) and 15 days (T15, **B**) of differentiation. [Ca^2+^] was assessed by measuring fura-2 ratios at 340 nm and 380 nm excitation as described in the Methods section. Representative traces of n > 6 experiments are shown. (**C**) Quantification of the Ca^2+^ peak responses induced by KCl in control and DM1 myotubes. (**D**) Quantification of the area under the curves (AUC). Data represent mean ± SD. *** *p* < 0.001 as compared to controls by Students’ *t* test.

First, to evaluate Ca^2+^ homeostasis in the DM1 cells we monitored depolarization-induced cytosolic Ca^2+^ signals and the size of the ER/SR Ca^2+^ store by imaging cells loaded with the ratiometric fluorescent Ca^2+^ sensitive probe Fura2-AM. During the maturation of control myotubes, we observed a threefold increase in the cytosolic Ca^2+^ response to KCl (peak Fura-2 340/380 nm ratios at T10: 0.94 ± 0.28; at T15: 3.12 ± 1.3, *p <* 0.001; Figure 1A–D), in line with the progression of differentiation [24]. In contrast, the KCl-induced Ca^2+^ signal in DM1 myotubes did not change between T10 and T15 of differentiation (peak Fura-2 340/380 nm ratios at T10: 0.67 ± 0.08; at T15: 0.38 ± 0.17; Figure 1C), and thus was significantly reduced at T15 as compared to controls (see Figure 1C). Similar reduction was observed in the area under the curves (AUC) at T15 of differentiation (control: 53.98 ± 13.96 *vs*. DM1: 26.04 ± 5.60; *p <* 0.001; Figure 1D), which represents the total amount of Ca^2+^ released from the ER/SR store during stimulation. Interestingly, the functional phenotype of mature DM1 myotubes is recapitulated in aged control myotubes, as indicated by reduced caffeine induced Ca^2+^ release (data not shown). These data indicate an impairment of Ca^2+^ release after depolarization, which might be due either to reduced size of the ER/SR Ca^2+^ store and/or impaired coupling-function of the Ca^2+^ signaling machinery in the sarcolemma or the ER/SR membrane.

**Figure 2:**
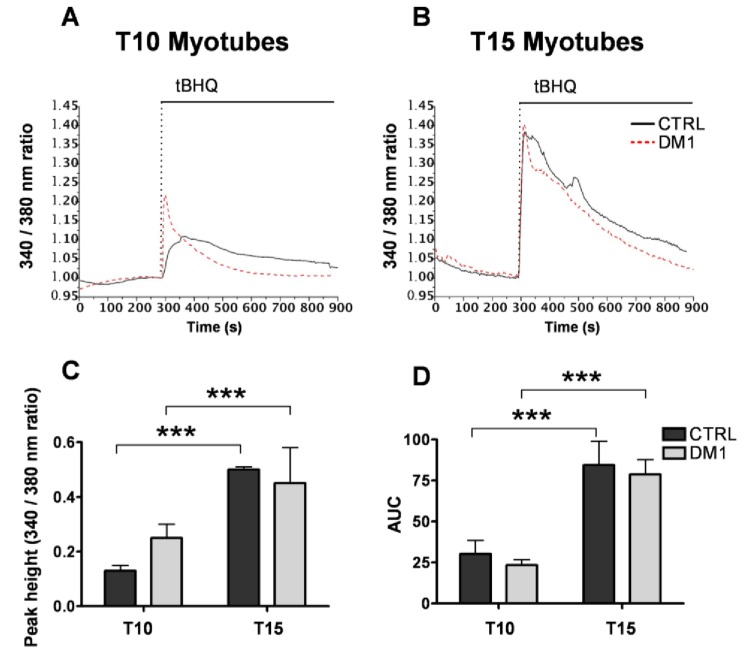
Size of the ER/SR Ca^2+^ stores in control and DM1 myotubes at progressive stages of differentiation. Depletion of ER/SR Ca^2+^ stores in myotubes at 10 (T10, **A**) and 15 days (T15, **B**) of differentiation was induced by the SERCA inhibitor tert-butylhydroquinone (tBHQ) at indicated time points. [Ca^2+^] was assessed by measuring Fura-2 ratios at 340 nm and 380 nm excitation as described in the Methods section. Representative traces of n > 8 experiments are shown. (**C**) Quantification of the Ca^2+^ peaks induced by tBHQ in control and DM1 myotubes. (**D**) Quantification of the area under the curves (AUC). Data represent mean ± SD. *** *p* < 0.001 as compared to controls by Students’ *t* test.

In order to discriminate between these possibilities, we directly measured the size of the ER/SR store by monitoring Ca^2+^ signals following the application of the SERCA inhibitor 2,5-di-(t-butyl)-hydroquinone (50 μM) to Fura-2-loaded control and DM1 myotubes. The inhibition of SERCA activity leads to complete release and depletion of the ER Ca^2+^ store, which allows to estimate its size by measuring the area under the recorded Ca^2+^ traces. Based on this method, there was no significant difference between control and DM1 myotubes both at 10 and 15 days of differentiation (Figure 2A–D). The size of ER/SR Ca^2+^ stores increased with maturation of both control and DM1 myotubes, thus DM1 phenotype is not caused by the depletion of Ca^2+^ stores. Therefore, impaired coupling between sarcolemmal Ca^2+^ channels and the RyRs, or their deficiency, is responsible for reduced Ca^2+^ signals induced by depolarization in DM1 myotubes. Accordingly, in the next set of experiments we investigated the expression pattern of the coupling machinery.

### 3.2. DM1 Myotubes and Muscle Show Aberrant Splicing of Ca^2+^ Transporters

**Figure 3:**
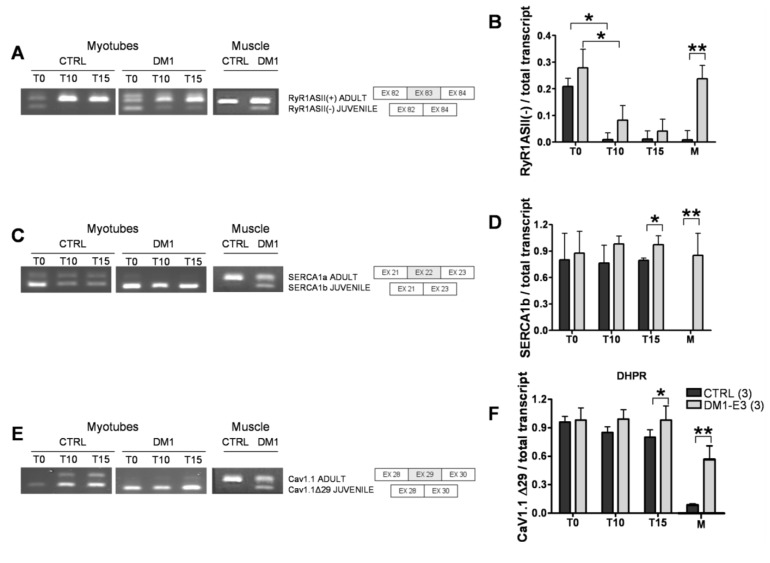
Splicing patterns of Ca^2+^ channels and transporters in control and DM1 myotubes at 10 (T10) and 15 (T15) days of differentiation. (**A**) RyR1 alternative splicing of fetal ASII(−) and ASII(+) isoforms and schematic representation of the ASII splicing isoforms of RyR1. (**B**) Quantification of RyR ASII(−) as compared to the total transcript. (**C**) SERCA1 alternative splicing of fetal SERCA1b and adult SERCA1a isoforms and schematic representation of the SERCA1 splicing isoforms. (**D**) Quantification of fetal SERCA1b as compared to the total transcript. (**E**) Cav1.1 alternative splicing of fetal Cav1.1Δ29 and adult Cav1.1 isoforms and schematic representation of the Cav1.1 splicing isoforms. (**F**) Quantification of fetal Cav1.1 Δ29 as compared to the total transcript. PCR products were separated in 3% agarose gel by electrophoresis. Data are the mean ± SD. * *p* < 0.05, ** *p* < 0.01 as compared to controls by Students’ *t* test. The numbers of single lines studied is given in brackets.

Expression of DMPK mRNAs containing CTG repeats interferes with the splicing of SERCA, RyR1 and Ca_v_1.1 in DM1 skeletal muscle [17,19,29], ensuing expression of fetal splice products, whose properties are not optimal for the function of adult muscle fibers and calcium balance.Therefore, we asked whether the splicing pattern of RyR1, SERCA 1 and Ca_v_1.1 were altered in T10 and T15 DM1 myotubes. In line with the observations of Kimura *et al.* [17], we found that the adult splice isoform RyR1 ASI(+) was not detectable in primary myoblasts/myotubes of both controls and DM1 samples (data not shown), indicating that the adult isoform ASI(+) was expressed only in the adult muscle. We then tested the amount of another variant ASII(−), which lacks ASII (exon 83, residues 3865–3870), dominantly expressed in human myotubes [17]. We determined that the amount of fetal isoform RyR1ASII(−) decreased in both control and DM1 samples during differentiation, indicating a gradual maturation of myotubes. However, the ASII(−) isoform was virtually absent in controls but still present in DM1 myotubes after 10 and 15 days of differentiation (Figure 3A,B).

Previous studies showed that splicing of SERCA1a (adult form) with exon 22 (SERCA1 +ex22) is repressed in DM1 patients [17,18,19] in favor of SERCA1b Δex22 (juvenile form). We performed the same protocols used for RyR1 to test the balance of SERCA1 splicing in differentiating muscle cultures from control and DM1 cell lines (Figure 3C,D). The amount of fetal SERCA1b Δex22 *vs.* total transcript decreased in controls but not in DM1 myotubes, where it was expressed and significantly different from controls at T15. 

DM1 is also associated with misregulated alternative splicing of CACNAIS, the gene encoding Ca_v_1.1 [19]. Skipping of exon 29 deletes a 19-amino acid fragment and determines the juvenile form of Ca_v_1.1. We assessed the regulation of Ca_v_1.1 splicing in primary muscle culture and found a progressive increase of ex29 inclusion in Ca_v_1.1 protein of T10 and T15 control myotubes. On the contrary, we found a near-complete skipping of ex29 in DM1 T10 and T15 myotubes which was significantly different from controls at T15 *( p <* 0.05) (Figure 3E,F).

The splicing pattern of the SERCA1, RyR1 and Ca_v_1.1 was assessed in the parental adult muscles from the same patients, from whom the muscle cells were established in culture. In line with the findings from *in vitro* experiments, the expression of fetal isoforms of SERCA1 and Cav1.1 was significantly higher in the DM1 adult muscle as compared to controls, showing a good correlation between *in vivo* and *in vitro* results. On the contrary, significantly higher level of RyR1 [Δ83] fetal variant was found only in the adult DM1 muscle samples, but not in the pathological DM1 myotubes. This difference could be due to the absence of innervation in our aneural muscle culture model, which otherwise leads to the consequent physiological adaptation of mature muscle fibers, together with RNA instability and the presence of other RyR1 splicing variants, such as RyR1 ASI(+), in adult muscle.

### 3.3. Expression and Subcellular Distribution of Ca^2+^ Transporters Is Altered in DM1 Myotubes

**Figure 4:**
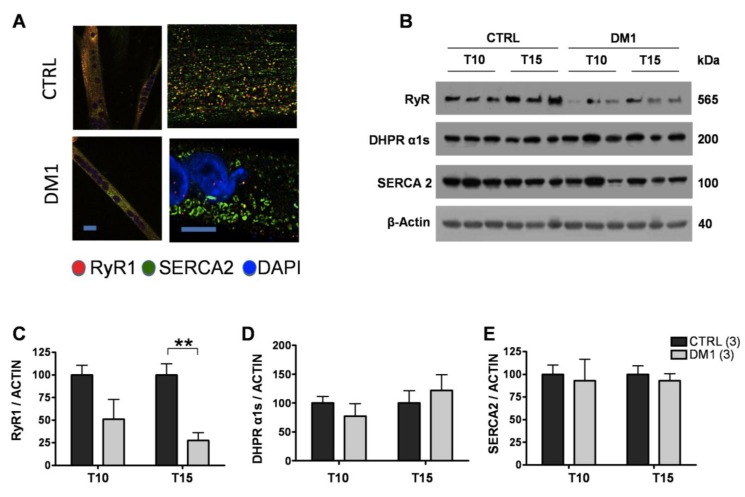
Expression of Ca^2+^ channels and transporters in control and DM1 myotubes at 10 (T10) and 15 (T15) days of differentiation. (**A**) Representative images of SERCA2 (green) and RyR1 (red), nuclei (blue-Dapi) immunofluorescence of T15 differentiated control and DM1 myotubes. Scale bar is 10 μm. (**B**) Representative Western blot analysis of RyR1, DHPR α1s (Cav1.1) and SERCA2 of control and DM1 myotubes at 10 and 15 days of differentiation. (**C**–**E**) Quantification of RyR1, DHPRα1s and SERCA2 protein levels normalized to β-actin. Data are the mean ± SD of two experiments. ** *p <* 0.01 as compared to controls by Students’ *t* test. The numbers of single lines studied is given in brackets.

Next, we analyzed the protein levels and subcellular distribution of SERCA and RyR1 in T15 myotubes of control and DM1 cells using immunofluorescence and Western blot analysis. Immunofluorescence analysis showed that RyR1 and SERCA2 had a similar diffuse localization in control myotubes (Figure 4A), while in DM1 myotubes RyR1 expression was substantially decreased, and showed an irregular, clustered pattern of distribution (Figure 4A). In line with the confocal images, Western blot analysis confirmed that the amount of RyR1 protein progressively decreased during DM1 maturation, at T10 being 51 ± 22% and at T15 27 ± 10% (*p <* 0.01) of relative controls. Importantly, we found no changes in the expression or subcellular distribution of SERCA2 and Cav1.1 (Figure 4B–E). These results indicate that, in addition to the aberrant splicing of mRNAs involved in ECC, the expression of RyR1 at the protein level is significantly reduced in DM1 myotubes, and its distribution on the ER/SR is markedly altered. These alterations account for the deregulated Ca^2+^ release from the ER/SR Ca^2+^ stores, and reflect a more general defect in ER/SR protein homeostasis.

### 3.4. The ER Stress Response Is Activated in DM1 Myotubes

Considering the aberrant splicing and expression of ER/SR proteins and the altered Ca^2+^ homeostasis, we tested whether the DM1 myotubes undergo ER stress, a concerted stress response to altered protein homeostasis in the organelle. Thus, we examined the expression of major ER stress markers at both the transcriptional and protein level.

**Figure 5:**
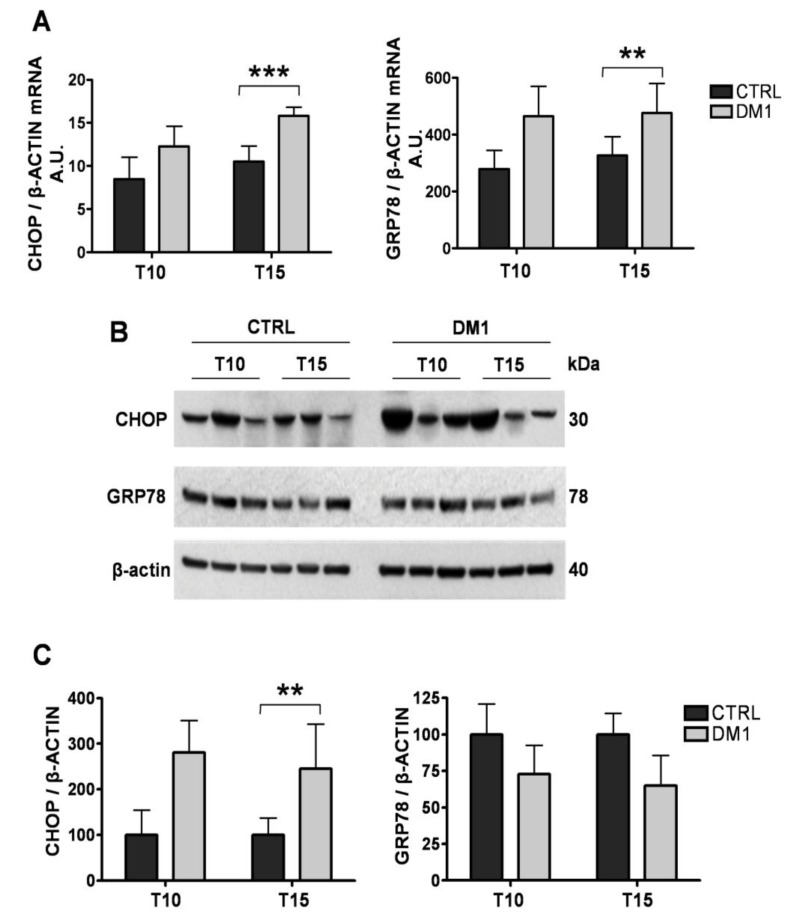
Characterization of the ER stress response in control and DM1 myotubes at 10 (T10) and 15 (T15) days of differentiation. (**A**) Expression levels of CHOP and GRP78 mRNA of control and DM1 myotubes at 10 and 15 days of differentiation as measured by quantitative RT-PCR. (**B**) Representative Western blot of CHOP and GRP78 from control and DM1 myotubes at 10 and 15 days of differentiation. (**C**) Quantification of CHOP and GRP78 protein levels, normalized to β-actin. The values represent the ratio of band intensities and are given in arbitrary units (A.U.) Data are expressed as mean ± SD of a single experiment of RT-PCR carried out in triplicate (**A**) and as mean ± ES of two experiments for WB (**C**). * *p* < 0.05, *** *p* < 0.001 as compared to controls by Students’ *t* test.

**Figure 6:**
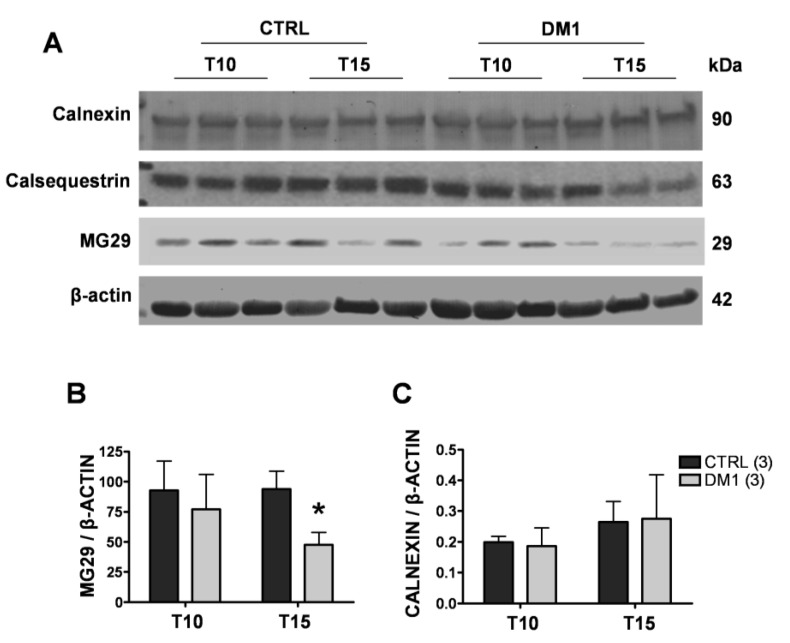
Expression of SR/ER resident proteins in control and DM1 myotubes at 10 (T10) and 15 (T15) days of differentiation. (**A**) Representative Western blot analysis of calnexin, calsequestrin and MG29 in control and DM1 myotubes at 10 and 15 days of differentiation. (**B**) Quantification of MG29 protein levels, normalized to β-actin. The values are given in arbitrary units (A.U.) and expressed as mean ± SD of two experiments. * *p <* 0.05 as compared to controls by Students’ *t* test. The numbers of single lines studied is given in brackets.

First, we quantified the mRNA of glucose regulated stress protein 78 (grp78) and C/EBP homologous protein (CHOP) by quantitative RT-PCR. As shown in Figure 5A, mRNA levels of the two proteins increased in DM1 myotubes after 10 days of differentiation, which became statistically significant at T15 for both grp78 (47%, *p <* 0.05) and CHOP (50%, *p <* 0.001) as compared to controls. Western blot analysis showed that CHOP levels were increased about twofold in DM1 myotubes as compared to controls at both T10 and T15 (Figure 5B,C), while no changes were observed in grp78 levels, probably indicating increased turnover of the protein (Figure 5B,C). In order to investigate the extent of deregulation of ER/SR protein homeostasis, we also examined the levels of major proteins involved in maintaining ER/SR structure and Ca^2+^ levels. While the levels of Ca^2+^ binding proteins calsequestrin and calnexin remained unchanged (Figure 6A,C), we found significantly decreased levels of MG29 in T15 DM1 myotubes, a protein implicated in the maintenance of triad junctions (Figure 6A,B).

These results confirmed that ER stress is already induced in early phases of differentiation in DM1 myotubes, and that the marked induction of the proapoptotic CHOP might be responsible for triggering cell death at T15 of differentiation, associated with the general derangement of ER/SR structure. 

## 4. Discussion

This study represents the first analysis of Ca^2+^ homeostasis and ER stress during maturation of DM1 muscle cells. We have chosen time points of 10 and 15 days of differentiation for the analysis, corresponding to the commencement of the previously observed apoptotic features (T10), and when the majority of myotubes displays features of full blown apoptosis (T15) [24]. 

We observed significantly decreased depolarization-induced Ca^2+^ release from the ER stores in mature T15, but not T10, DM1 myotubes as compared to controls (see Figure 1). Interestingly, the altered Ca^2+^ response occurred in concomitance with the increased ROS and programmed cells-death [24]. The reduction of Ca^2+^ release was not caused by a depletion of ER Ca^2+^ stores, since the amount of Ca^2+^ available for the release by ECC in T15 DM1 myotubes was found to be normal (see Figure 2). In contrast, the phenotype was associated with the following abnormalities: (i) a predominant expression of immature splice products of genes involved in Ca^2+^ transport and channeling, (see Figure 3); (ii) significantly reduced levels of RyR1 protein, that decreased progressively from T10 to T15 in DM1 myotubes (Figure 4); (iii) induction of ER stress markers and a progressive membrane deformation that may induce disruption of triad junction and SR network (Figure 5, Figure 6) [30].

We found that both fetal SERCA1b Δex22 and fetal Cav1.1 Δex29 were significantly increased in T15 DM1 myotubes as compared to controls, where the proportion of adult isoforms of SERCA (ex22) and Cav1.1 (ex29) increased with the progression of the *in vitro* muscle maturation. In the same cellular model (T10 and T15 DM1 myotubes) we previously described a splicing unbalance of MBNL1 and Insulin Receptor and nuclear accumulation of CUG-containing *DMPK* mutant transcripts (foci) [24], strongly suggesting that the size of CTG repeats in the *DMPK* gene plays a role in determining the splicing unbalance of all analyzed genes.

Aberrant splicing of RyR1, SERCA1 and Cav1.1, has been well documented in adult DM1 muscle [17,19,29], but the data relating to the *in vitro* muscle maturation were missing. The splicing pattern of SERCA1-ex22 and Cav1.1-ex29 has a similar developmental window [19], and in both cases is regulated by cis-acting elements specific for MBNL1 binding [18,19]. Recently, it was reported that the splicing regulation of SERCA1a (+ex22) and SERCA1b (Δex22) is mediated by protein kinase C (PKC) [31]. No functional difference has been described between the SERCA 1a and 1b isoforms, which differ in their *C*-terminal region [32] in COS-1 cells. However, it is possible that the sequence difference between the isoforms provides binding sites or affects the binding to muscle specific factors absent in COS cells which influence their ATPase activity. Cav1.1 plays a key role in EC coupling. The skipping of exon 29 deletes a 19-amino acid fragment within the IVS3-IVS4 extracellular loop, adjacent to the IVS4 voltage sensor and determines the fetal form of Cav1.1. Notably, the expression of fetal Cav 1.1 Δex29 was significantly correlated with weakness in DM1 patients [19]. Quantitative analysis revealed that the fetal Cav1.1 Δex29 channel triggers a larger evoked Ca^2+^ transient in adult muscle [19], supporting a gain-of-function increase in depolarization-induced Ca^2+^ transients, that may be further amplified by local Ca^2+^-dependent activation of RyR1 within the triad junction. Increased ex29 skipping and enhanced Cav1.1 gating may explain the augmented nifedipine-sensitive Ca^2+^ influx in DM1 myotubes [33]. The combined effect of increased ECC activity coupled with myotonia, due to mis-splicing of a chloride channel [4,26], and altered Ca^2+^ homeostasis (RyR1 and SERCA1 mis-splicing) may lead to a chronic Ca^2+^ overload already observed in DM1 myotubes [33].

Immunoblot analysis determined the quantity of RyR1, SERCA2 and Cav1.1 protein expression in T10 and T15 DM1 myotubes. Whilst SERCA1 is the predominant isoform in skeletal muscle, SERCA2 is more widely expressed, thus can be found, both in myoblasts and myotubes. Thus, in order to assess the role of this transporter during the progress of the DM1 phenotype we have analyzed the expression and localization of SERCA2. Our results indicate that the splicing defect observed in the SERCA1 gene is accompanied by changes in the localization of SERCA2 expression. We observed normal protein levels of SERCA2 and Cav1.1 and a significant decrease of RyR1 in T15 affected muscle cells. The reduction of RyR1 can explain the significantly reduced mobilization of Ca^2+^ from the ER/SR store and could be the consequence of the accumulation of excess Ca^2+^ in the microdomain and consequent activation of Ca^2+^-dependent proteases [22]. Previous studies on Ca^2+^ related proteins were inconclusive, suggesting, from one side, a decrease in the total amount of SERCA protein in cultured DM1 myotubes [20], and from another an increase of SERCA 1 expression in DM1 skeletal muscle samples [34]. Moreover, Kimura *et al.* reported a trend towards increased protein levels of SERCA1 and 2 in the ER fraction of DM1 mouse, but not of the RyR1 [17]. These discordant results may be accounted for the different models used (human [34] or mouse model [17], or by the different analyses of protein quantification (WB *vs*. Ca^2+^-dependent phosphorylation and ELISA [20]). Taken together, our data clearly indicate that the mis-regulated splicing events lead to the production of proteins with altered properties, and suggest that the deregulation of Ca^2+^ homeostasis in myotonic dystrophy could be determined by the failure to complete the developmental switch to a specific set of alternatively spliced genes, resulting in insufficient Ca^2+^ release from the ER/SR due to the reduction of functional RyR1 protein levels. It has been previously reported that increased ROS levels can trigger increased opening probability of the RyR [35], which might compensate the DM1 phenotype. However, in the present study we have no evidence that oxidative stress indeed occurs at the level of RyR1 receptors, and we assume that in the presence of the deranged coupling apparatus the relatively modest increase of open probability of the RyR1 channels would not fully compensate the almost 90% reduction of depolarization induced Ca^2+^ signals (See Figure 1).

The long-term consequence of deregulated protein synthesis can be linked to apoptotic events through the observed ER stress response. The kinetics of cellular responses to ER stress can typically be divided in two phases. First, adaptation (the so-called unfolded protein response) aims to re-establish normal protein folding in the ER by up-regulating key chaperones such as grp78, and to provide the energetic basis for cell survival by up-regulating mitochondrial function [36]. In the case of fervent or persistent ER stress, exhaustion of the adaptation mechanisms occurs, triggering cell death either by direct Ca^2+^ transfer to the mitochondria [37], or by inducing a pro-apoptotic transcriptional response, often mediated by CHOP [38]. Here, we observed progressive up-regulation of CHOP at both the mRNA and protein level in DM1 myotubes, which became significant at T15 of differentiation, in line with our previous observations of increased apoptosis at this stage. Interestingly, while grp78 was induced at the transcriptional level, it did not result in increased protein expression. This was not due to a general loss of the ER/SR, since both the level of SERCA and the ER Ca^2+^ binding proteins calsequestrin and calnexin remained unchanged in DM1 myotubes. Moreover, the overall structure of the ER/SR has not changed drastically as judged by the distribution of SERCA on the immunofluorescence images. Thus, the missing accumulation of grp78 protein is most likely the result of increased turnover of the protein, probably mediated by the ER-associated degradation system [39]. Our findings confirm previous observations: ER stress-related molecules were observed in DM1 muscle [9] in relation to apoptotic nuclei and the apoptotic executors, the caspase-3 and 7 [9]. Most importantly, while the role of cell damage mediated by the ER stress has been recognized in a wide range of diseases (e.g., neurodegeneration or liver disease), our study represents a novel example of recognizing this important pathologic mechanism in the context of genetic muscular disease [40]. Data regarding the reduction of MG29 are consistent with the model of membrane deformation and subsequent disruption of triad junctions, a condition that mimics the aging of muscle, and altogether in line with previous findings showing the alteration of T tubules related to DMPK [41] and BIN1-E11 mis-splicing [42].

## 5. Conclusions

In summary, our data suggest a general pathologic mechanism in DM1 muscle, in which mis-splicing of pre-mRNAs of genes involved in muscle Ca^2+^ handling, coupled to deregulated Ca^2+^ homeostasis and excitation-contraction coupling, leads to muscle weakness by ER-stress and consequent apoptosis.

## References

[1] Harper P.S. (2001). Myotonic Dystrophy.

[2] Wagner A., Steinberg H. (2008). Hans Steinert (1875–1911). J. Neurol..

[3] Machuca-Tzili L., Brook D., Hilton-Jones D. (2005). Clinical and molecular aspects of the myotonic dystrophies: A review. Muscle Nerve.

[4] Wheeler T.M., Thornton C.A. (2007). Myotonic dystrophy: RNA-Mediated muscle disease. Curr. Opin. Neurol..

[5] Cooper T.A. (2009). Chemical reversal of the RNA gain of function in myotonic dystrophy. Proc. Natl. Acad. Sci. USA.

[6] Brouwer J.R., Willemsen R., Oostra B.A. (2009). Microsatellite repeat instability and neurological disease. Bioessays.

[7] Gomes-Pereira M., Monckton D.G. (2006). Chemical modifiers of unstable expanded simple sequence repeats: What goes up, could come down. Mutat. Res..

[8] Thornell L.E., Lindstom M., Renault V., Klein A., Mouly V., Ansved T., Butler-Browne G., Furling D. (2009). Satellite cell dysfunction contributes to the progressive muscle atrophy in myotonic dystrophy type 1. Neuropathol. Appl. Neurobiol..

[9] Ikezoe K., Nakamori M., Furuya H., Arahata H., Kanemoto S., Kimura T., Imaizumi K., Takahashi M.P., Sakoda S., Fujii N. (2007). Endoplasmic reticulum stress in myotonic dystrophy type 1 muscle. Acta Neuropathol..

[10] Timchenko N.A., Cai Z.J., Welm A.L., Reddy S., Ashizawa T., Timchenko L.T. (2001). RNA CUG repeats sequester CUGBP1 and alter protein levels and activity of CUGBP1. J. Biol. Chem..

[11] Miller J.W., Urbinati C.R., Teng-Umnuay P., Stenberg M.G., Byrne B.J., Thornton C.A., Swanson M.S. (2000). Recruitment of human muscleblind proteins to (CUG)(n) expansions associated with myotonic dystrophy. EMBO J..

[12] Botta A., Vallo L., Rinaldi F., Bonifazi E., Amati F., Biancolella M., Gambardella S., Mancinelli E., Angelini C., Meola G. (2007). Gene Expression analysis in myotonic dystrophy: Indications for a common molecular pathogenic pathway in DM1 and DM2. Gene Expr..

[13] Fanchaouy M., Polakova E., Jung C., Ogrodnik J., Shirokova N., Niggli E. (2009). Pathways of Abnormal Stress-Induced Ca^2+^ Influx into Dystrophic Mdx Cardiomyocytes. Cell Calcium.

[14] Szabadkai G., Duchen M.R. (2008). Mitochondria: The Hub of cellular Ca^2+^ signaling. Physiology (Bethesda).

[15] Bernardi P. (1999). Mitochondrial transport of cations: Channels, exchangers, and permeability transition. Physiol. Rev..

[16] Cherednichenko G., Hurne A.M., Fessenden J.D., Lee E.H., Allen P.D., Beam K.G., Pessah I.N. (2004). Conformational activation of Ca^2+^ entry by depolarization of skeletal myotubes. Proc. Natl. Acad. Sci. USA.

[17] Kimura T., Nakamori M., Lueck J.D., Pouliquin P., Aoike F., Fujimura H., Dirksen R.T., Takahashi M.P., Dulhunty A.F., Sakoda S. (2005). Altered mRNA splicing of the skeletal muscle ryanodine receptor and sarcoplasmic/endoplasmic reticulum Ca^2+^-ATPase in myotonic dystrophy type 1. Hum. Mol. Genet..

[18] Hino S., Kondo S., Sekiya H., Saito A., Kanemoto S., Murakami T., Chihara K., Aoki Y., Nakamori M., Takahashi M.P. (2007). Molecular mechanisms responsible for aberrant splicing of SERCA1 in myotonic dystrophy type 1. Hum. Mol. Genet..

[19] Tang Z.Z., Yarotskyy V., Wei L., Sobczak K., Nakamori M., Eichinger K., Moxley R.T., Dirksen R.T., Thornton C.A. (2012). Muscle weakness in myotonic dystrophy associated with misregulated splicing and altered gating of Ca(V)1.1 calcium channel. Hum. Mol. Genet..

[20] Benders A.A., Timmermans J.A., Oosterhof A., Ter Laak H.J., van Kuppevelt T.H., Wevers R.A., Veerkamp J.H. (1993). Deficiency of Na+/K(+)-ATPase and sarcoplasmic reticulum Ca(^2+^)-ATPase in skeletal muscle and cultured muscle cells of myotonic dystrophy patients. Biochem. J..

[21] Kimura T., Takahashi M.P., Okuda Y., Kaido M., Fujimura H., Yanagihara T., Sakoda S. (2000). The expression of ion channel mRNAs in skeletal muscles from patients with myotonic muscular dystrophy. Neurosci. Lett..

[22] Kimura T., Lueck J.D., Harvey P.J., Pace S.M., Ikemoto N., Casarotto M.G., Dirksen R.T., Dulhunty A.F. (2009). Alternative splicing of RyR1 alters the efficacy of skeletal EC coupling. Cell Calcium.

[23] Futatsugi A., Kuwajima G., Mikoshiba K. (1995). Tissue-Specific and developmentally regulated alternative splicing in mouse skeletal muscle ryanodine receptor mRNA. Biochem. J..

[24] Loro E., Rinaldi F., Malena A., Masiero E., Novelli G., Angelini C., Romeo V., Sandri M., Botta A., Vergani L. (2010). Normal myogenesis and increased apoptosis in myotonic dystrophy type-1 muscle cells. Cell Death Differ..

[25] Botta A., Bonifazi E., Vallo L., Gennarelli M., Garre C., Salehi L., Iraci R., Sansone V., Meola G., Novelli G. (2006). Italian guidelines for molecular analysis in myotonic dystrophies. Acta Myol..

[26] Botta A., Rinaldi F., Catalli C., Vergani L., Bonifazi E., Romeo V., Loro E., Viola A., Angelini C., Novelli G. (2008). The CTG repeat expansion size correlates with the splicing defects observed in muscles from myotonic dystrophy type 1 patients. J. Med. Genet..

[27] Barreto-Chang O.L., Dolmetsch R.E. (2009). Calcium imaging of cortical neurons using Fura-2. AM. J. Vis. Exp..

[28] Brini M., de Giorgi F., Murgia M., Marsault R., Massimino M.L., Cantini M., Rizzuto R., Pozzan T. (1997). Subcellular analysis of Ca^2+^ homeostasis in primary cultures of skeletal muscle myotubes. Mol. Biol. Cell.

[29] Lin X., Miller J.W., Mankodi A., Kanadia R.N., Yuan Y., Moxley R.T., Swanson M.S., Thornton C.A. (2006). Failure of MBNL1-Dependent post-natal splicing transitions in myotonic dystrophy. Hum. Mol. Genet..

[30] Weisleder N., Brotto M., Komazaki S., Pan Z., Zhao X., Nosek T., Parness J., Takeshima H., Ma J. (2006). Muscle aging is associated with compromised Ca^2+^ spark signaling and segregated intracellular Ca^2+^ release. J. Cell Biol..

[31] Zhao Y., Koebis M., Suo S., Ohno S., Ishiura S. (2012). Regulation of the alternative splicing of sarcoplasmic reticulum Ca(2)(+)-ATPase1 (SERCA1) by Phorbol 12-Myristate 13-Acetate (PMA) via a PKC pathway. Biochem. Biophys. Res. Commun..

[32] Maruyama K., MacLennan D.H. (1988). Mutation of aspartic Acid-351, Lysine-352, and Lysine-515 alters the Ca^2+^ transport activity of the Ca^2+^-ATPase expressed in COS-1 Cells. Proc. Natl. Acad. Sci. USA.

[33] Jacobs A.E., Benders A.A., Oosterhof A., Veerkamp J.H., van Mier P., Wevers R.A., Joosten E.M. (1990). The calcium homeostasis and the membrane potential of cultured muscle cells from patients with myotonic dystrophy. Biochim. Biophys. Acta.

[34] Damiani E., Angelini C., Pelosi M., Sacchetto R., Bortoloso E., Margreth A. (1996). skeletal muscle sarcoplasmic reticulum phenotype in myotonic dystrophy. Neuromuscul. Disord..

[35] Pessah I.N. (2001). Ryanodine receptor acts as a sensor for redox stress. Pest. Manag. Sci..

[36] Bravo R., Vicencio J.M., Parra V., Troncoso R., Munoz J.P., Bui M., Quiroga C., Rodriguez A.E., Verdejo H.E., Ferreira J. (2011). Increased ER-Mitochondrial coupling promotes mitochondrial respiration and bioenergetics during early phases of ER stress. J. Cell Sci..

[37] Chami M., Oules B., Szabadkai G., Tacine R., Rizzuto R., Paterlini-Brechot P. (2008). Role of SERCA1 truncated isoform in the proapoptotic calcium transfer from ER to mitochondria during ER stress. Mol. Cell.

[38] Tabas I., Ron D. (2011). Integrating the mechanisms of apoptosis induced by endoplasmic reticulum stress. Nat. Cell Biol..

[39] Smith M.H., Ploegh H.L., Weissman J.S. (2011). Road to ruin: Targeting proteins for degradation in the endoplasmic reticulum. Science.

[40] Rayavarapu S., Coley W., Nagaraju K. (2012). Endoplasmic reticulum stress in skeletal muscle homeostasis and disease. Curr. Rheumatol. Rep..

[41] Ueda H., Shimokawa M., Yamamoto M., Kameda N., Mizusawa H., Baba T., Terada N., Fujii Y., Ohno S., Ishiura S. (1999). Decreased expression of myotonic dystrophy protein kinase and disorganization of sarcoplasmic reticulum in skeletal muscle of myotonic dystrophy. J. Neurol. Sci..

[42] Fugier C., Klein A.F., Hammer C., Vassilopoulos S., Ivarsson Y., Toussaint A., Tosch V., Vignaud A., Ferry A., Messaddeq N. (2011). Misregulated alternative splicing of BIN1 is associated with T tubule alterations and muscle weakness in myotonic dystrophy. Nat. Med..

